# Skin reaction to inhaled tiotropium bromide: a case report

**DOI:** 10.1186/1752-1947-5-119

**Published:** 2011-03-28

**Authors:** Cristoforo Incorvaia, Nicola Fuiano, Raffaella Megali, Gian Galeazzo Riario-Sforza

**Affiliations:** 1 Department of Allergy/Pulmonary Rehabilitation, ICP Hospital, Milan, Italy

## Abstract

**Introduction:**

Systemic reactions to inhaled drugs are rare. To the best of our knowledge, we report the first case of generalized itching related to the use of tiotropium bromide, a long acting inhaled anti-cholinergic agent commonly used to treat chronic obstructive pulmonary disease.

**Case presentation:**

A 78-year-old Caucasian woman was referred to our facility for allergological evaluation. Our patient had been treated twice with tiotropium for chronic obstructive pulmonary disease and had experienced an allergic reaction with itching. We performed a double-blind placebo-controlled inhalation challenge for our patient with tiotropium and a placebo. Inhalation tests yielded positive results for tiotropium and negative results for the placebo. The results of a skin prick test with tiotropium were negative.

**Conclusions:**

These findings reveal that tiotropium may elicit immediate skin allergic reactions. The negative result from the skin test suggests that such a reaction is not immunoglobulin E-mediated.

## Introduction

Tiotropium bromide is a long-acting inhaled anti-cholinergic agent commonly used to treat chronic obstructive pulmonary disease (COPD) [1]. A recently published review on drug safety data obtained from 26 clinical trials involving approximately 17,000 patients reported that no difference was observed between patients treated with tiotropium bromide and those given a placebo, with respect to the rates of adverse events caused by cardiac, vascular, nervous, and lower respiratory disorders [2]. Instead, adverse events caused by the anti-cholinergic effect of tiotropium were much more common than those due to the placebo. These adverse effects included dryness in the mouth (observed in about 16% of the patients treated), constipation, dyspepsia, gastroesophageal reflux, dysuria, and urinary retention. The information provided on skin reactions is puzzling; in the patient information sheet for tiotropium the 'Possible side effects' section reports: 'Allergic reactions. Symptoms may include: itching, rash, swelling of the lips, tongue, or throat (trouble swallowing)'. However, these reactions have not been mentioned in the safety data review [2]. In addition, on performing a systematic literature search of the MEDLINE and EMBASE databases, we found only one study reporting a tiotropium-associated skin reaction; this study described a photosensitive lichenoid eruption in a 72-year-old man [3]. Here, we report a case where a skin reaction to tiotropium was obtained in a double-blind placebo-controlled challenge.

## Case presentation

Our patient was a 78-year-old Caucasian woman with COPD for approximately 30 years who had only been treated for it recently. In June 2009, our patient was referred to a pneumologist who prescribed the use of an 18 μg tiotropium inhalation capsule once daily; this prescription was based on a forced expiratory volume in one second (FEV_1_) value of 59.7% that had been measured using spirometry. Our patient was not under drug treatment for any other disease. After three days, she developed generalized itching with no wheals or edema. The family physician stopped the tiotropium treatment and prescribed inhaled salbutamol as needed for dyspnea. After five months, our patient was referred to another pneumologist because of exacerbation of her COPD symptoms; however, she did not inform the pneumologist of her previous skin reaction to tiotropium. A FEV_1 _value of 56.4% was obtained using spirometry, and tiotropium was again prescribed. This time, our patient experienced generalized itching approximately one hour after the first dose. Our patient's family physician referred her to us for allergological evaluation. We performed a double-blind placebo-controlled inhalation challenge with tiotropium, administering the drug or the placebo on two days consecutively. The lactose monohydrate excipient contained in the powder was used as the placebo. Our patient experienced generalized itching 50 minutes after inhaling tiotropium but had no symptoms after inhaling the placebo. As our patient had experienced an immediate allergic reaction, we performed a skin prick test (SPT) with tiotropium diluted in physiological solution; however, the SPT result was negative. In addition, SPTs performed using a standard panel of environmental allergens yielded negative results. We advised our patient to avoid using tiotropium in the future and prescribed a 9 μg formoterol inhalation capsule twice a day; this medication was well tolerated and effective.

## Conclusions

Skin reactions to inhaled drugs used to treat COPD and asthma are rare and mostly concern corticosteroids [4]. Specifically, no reaction with generalized itching associated with the use of tiotropium bromide has been reported. The repeated reaction with itching in our patient led us to perform a double-blind, placebo-controlled challenge, which revealed that tiotropium may elicit immediate skin allergic reactions. The negative result from the skin test suggests that such a reaction is not mediated by immunoglobulin E.

## Consent

Written informed consent was obtained from the patient for publication of this case report and any accompanying images. A copy of the written consent is available for review by the Editor-in-Chief of this journal.

## Competing interests

The authors declare that they have no competing interests.

## Authors' contributions

CI analyzed and interpreted the data from our patient regarding the results of challenge test and skin tests and wrote the manuscript. RM performed the challenge test. GRS performed the skin tests. NF was a major contributor in writing the manuscript. All authors read and approved the final manuscript.
